# Evaluation of Angiopoietins 1 and 2 in Malaria-Infested Children

**DOI:** 10.1155/2020/2169763

**Published:** 2020-05-26

**Authors:** A. O. Oluboyo, S. I. Chukwu, B. O. Oluboyo, O. O. Odewusi

**Affiliations:** Department of Medical Laboratory Science, College of Medicine and Health Sciences, Afe Babalola University, Ado Ekiti, Ekiti State, Nigeria

## Abstract

**Background:**

Malaria could affect people of all ages, most especially young children. The study evaluated the levels of serum angiopoietin-1 (Ang-1) and angiopoietin-2 (Ang-2) which are critical regulators of endothelial activation and integrity with some hematological parameters (total white blood cell counts (WBC), total red blood cell counts (RBC), platelet counts, and malaria parasite density) in malaria-infected children.

**Method:**

A total of 92 blood samples from children between the ages of 6 months to 15 years were analyzed. The samples consisted of 30 cases of severe malaria, 40 cases of uncomplicated malaria, and 22 apparently healthy subjects served as control. Serum Ang-1 and -2 levels were determined using the enzyme-linked immunosorbent assay technique. The hematological parameters were determined using the WHO standard.

**Results:**

There was significant decrease (*p* < 0.05) in serum Ang-1 of uncomplicated malaria and severe malaria compared with the control, while significant increase (*p* < 0.05) was observed in Ang-2 and Ang-2/Ang-1 ratio in uncomplicated malaria and severe malaria compared with the control. RBC and platelet showed significant decrease, while WBC showed significant increase in severe malaria compared with uncomplicated malaria and control.

**Conclusion:**

This study showed that subjects with malaria infection had a significant increase of Ang-2 and Ang-2 : Ang-1 ratio but presented with a significant decrease of Ang-1. Ang-1 and Ang-2 may be used to determine the severity of malaria infection since their levels differ significantly in malaria subjects compared with the control.

## 1. Introduction

Malaria is a common life-threatening disease which is of high prevalence in both tropical and subtropical regions [1]. Malaria could affect people of all ages, most especially young children. *Plasmodium falciparum* is the most common species of Plasmodium causing malaria infection in Africa, and it is the most aggressive species of Plasmodium often killing people by coma or anaemia [2]. Malaria affects people of all sex and ages, but the most vulnerable people are young children, pregnant women, HIV/AIDS patients, and international travellers from nonendemic areas [3]. Among the risk groups, young children particularly between 6 months and 5 years of age in endemic regions of Africa, where nearly a quarter of all childhood deaths are due to malaria, are most susceptible to severe malaria and contribute to a bulk of malaria deaths worldwide [4, 5]. Malaria infection occurs as either uncomplicated disease or as a severe disease [6]. These two forms of malaria can be differentiated with the help of a careful assessment of the patient suspected positive for malaria. Consequently, it is important to carefully distinguish between acute uncomplicated malaria and severe malaria as this can affect the treatment and prognostic implications of such disease and thereby lead to further complications [7].

### 1.1. Angiopoietin-1

Angiopoietins are ligands of the Tie-2 receptor which are expressed on the endothelial cells and regulate endothelial quiescence during normal physiological function. They are proteins that are closely involved in the stability of the endothelium, and their levels are altered in various inflammatory conditions mostly due to endothelial activation [8, 9]. Under normal physiological conditions, higher levels of Ang-1 which are stored in the platelets promote quiescence in the vascular endothelium. In *Plasmodium falciparum* malaria, the production of angiogenic factors is principally associated with an increase in the cytoadherence of infected erythrocytes to the vascular endothelium [10].

### 1.2. Angiopoietin-2

Ang-2 is a secreted glycoprotein that regulates vascular remodeling by promoting endothelial cell survival, proliferation, and migration and also helps in destabilizing the interaction between endothelial cells and perivascular cells. It is stored in the Weibel–Palade bodies together with von Willebrand factor and is rapidly mobilized and released into the blood stream during endothelial activation [11]. Ang-2 is thought to amplify Ang-1 activation by opposing the effects of Ang-1 [12]. In the presence of Ang-1, Ang-2 acts as a functional antagonist, and the Ang-2-Tie-2 interaction results in the blocking of the protective, anti-inflammatory, and antiapoptotic effect of Ang-1 [13].

### 1.3. Interaction of Angiopoietins 1 and 2 during Malaria Infection

During infection, there is an abnormal adherence (cytoadhesion) of red blood cells infected by Plasmodium to the endothelium which is one of the determining factors for the severity and evolution of the disease [14]. During inflammation or on exposure to plasmodium-infected red blood cells, angiopoietin-2 which is stored together with von Willebrand factor in Weibel–Palade bodies is rapidly mobilized and enormously released into the blood stream [11]. The red cell alterations may also be caused by thrombophilic factors and platelet abnormalities and may also result in vascular thrombosis [15]. Angiopoietin-2 then binds to the Tie-2 receptor and promotes proinflammatory and prothrombotic pathways as well as microvascular leak, thereby interfering with constitutive angiopoietin-1 (Ang-1)/Tie signalling in endothelial cells. Angiopoietin dysregulation indicates a change in the normally low Ang-2 : Ang-1 ratio either as a result of increased angiopoietin-2 or decreased angiopoietin-1 or even a combination of the two conditions [16]. Therefore, since Angiopoietin-1 and angiopoietin-2 are critical regulators of endothelial activation and integrity, their levels in serum samples of malaria subjects may help to discriminate between severe and uncomplicated malaria. Therefore, the study sets are established to evaluate the levels of serum angiopoietin-1 and angiopoietin-2 with some hematological parameters (total white blood cell counts, total red blood cell counts, platelet counts, and malaria parasite density) in malaria-infected children.

## 2. Materials and Methods

The study investigated a total of 92 subjects (6 months–15 years) using a stratified sampling method in subjects who were admitted at the Federal Teaching Hospital, Ido-Ekiti, Ekiti State, for malaria infection between March and June 2018. Ethical approval was also collected from the Federal Medical Center, Ido-Ekiti, and and College of Medicine and Health Sciences, Afe babalola University, Ado Ekiti, before sample collection. Blood samples were collected from children who were positive for *Plasmodium falciparum* malaria only after their parents voluntarily gave informed consent. All sample analyses were carried out at the Laboratory facility of the Medical Laboratory Science Department, Afe-Babalola University, Ado-Ekiti, Ekiti State. Out of the 92 children recruited as test subjects, 30 had severe malaria (based on clinical manifestations), 40 had uncomplicated malaria, and 22 were control subjects. The children were also grouped into three groups based on age range (0–5, 6–10, and 11–15).

About 3 ml of blood sample was collected from each volunteer subject. 1 ml of blood sample was dispensed into a 1 ml EDTA sample bottle containing 10 *μ*l of EDTA, while the remaining blood (2 ml) was dispensed into a plain sample bottle. The blood sample in the EDTA sample bottle was used to detect the presence of malaria parasite using a thick blood film, and a thin film was prepared to identify the species of Plasmodium causing the infection. Thereafter, white blood cell count (WBC), red blood cell count (RBC), and platelet count were estimated along with malaria parasite density. The blood sample in the plain bottles was allowed to clot, retracted, and centrifuged for 5 minutes at 3000 revolutions per minute (RPM) after which the serum was separated from the red cell, transferred into another plain tube, and stored at −20°C until needed for the analysis of angiopoietin-1 and angiopoietin-2.

### 2.1. Methods of Analysis

Detection of malaria parasites: the diagnosis of malaria was performed using both thick and thin films [17–19]. WBC, RBC, and platelet counts were estimated using manual methods [20] and parasite density counts using WHO's recommendation [21]. Angiopoietin-1 and -2 in serum were determined using enzyme-linked immunosorbent assay (ELISA) using Melsin Medical Co., Limited, China [22]. The samples were diluted (1 in 5) during analysis with the sample diluent provided by the manufacturer.

### 2.2. Statistical Analysis

Data collected were subjected to statistical analysis using Statistical Package for Social Sciences (SPSS) version 23. Values were expressed as mean ± standard deviation (SD). Analysis of variance (ANOVA) was used to determine the level of significance. *p* < 0.01, and *p* < 0.05 was considered statistically significant. Receiver operating characteristic (ROC) analysis was also carried out.

## 3. Results

The results of WBC, RBC, platelet, and angiopoietin-1 and -2 levels are presented in tables as follows.

Table 1 shows the mean ± SD of angiopoietin levels, WBC, RBC, and platelet counts in malaria and control subjects. There was a significance difference (*p* < 0.01 and *p* < 0.05) observed when the parameters in malaria subjects were compared with control subjects. A significant decrease was seen between the Ang-1 levels of healthy control, uncomplicated malaria, and severe malaria subjects; however, a significant increase was seen between Ang-2 levels of healthy control, uncomplicated malaria, and severe malaria subjects. Also, the ratio of Ang-2 : Ang-1 showed a significant increase (*p* < 0.05) between severe malaria subjects and uncomplicated malaria subjects and also with control subjects but not between uncomplicated malaria and control participants.

Table 2 shows the WBC, RBC, platelet, MP density, and angiopoietin level of uncomplicated malaria based on age groups. There was no significant difference observed in the parameters.

Table 3 shows the WBC, RBC, platelet, MP density, and angiopoietin level of severe malaria based on age groups. There were significant increases (*p* < 0.01) observed in the levels of Ang-1 and Ang-2 based on age groups.

Table 4 shows the ROC analysis to assess the parameters' ability to serve as markers in both severe and uncomplicated malaria. The area under the ROC curve shows that the WBC, RBC, platelet, MP density, angiopoietin-2, and Ang-2/Ang-1 have significant ability (*p* < 0.001) to discriminate the severity of malaria infection.

## 4. Discussion

In this study, a significant decrease was observed between levels of Ang-1 in severe and healthy control subjects and also between uncomplicated malaria and healthy control subjects but not between severe and uncomplicated malaria subjects. The results of this study are similar to the study conducted by some researchers [23] which showed higher levels of Ang-1 in healthy control subjects compared with severe malaria and uncomplicated malaria and a significant decrease between Ang-1 levels in severe and uncomplicated malaria compared with that of control subjects.

A significant increase was also observed in the levels of Ang-2 among severe malaria and uncomplicated malaria compared with the control group. Ang-2 was elevated in severe malaria patients compared with that of uncomplicated malaria and healthy controls across all ages and age groups. These findings are similar to a work which stated that Ang-2 levels were higher in Indonesian adults with severe malaria [10]. Variations in Ang-2 levels were observed as better predictors of death than other disease markers such as lactate. It is also consistent with another study in Malawian children where it was reported that higher levels of Ang-2 was seen in children with severe malaria compared with those with uncomplicated malaria [24]. Therefore, our findings, compared with that obtained from previous reports, suggest that Ang-2 is a quantifiable and unbiased marker of malaria severity. Although this study did not report whether Ang-1 and Ang-2 could be used to predict which of the children with uncomplicated malaria is slowly progressing to severe malaria, the results obtained, however, evidently demonstrated that reduced Ang-1 and increased Ang-2 levels are sensitive and specific signs of severe malaria disease that effectively differentiate between uncomplicated malaria and severe malaria. Therefore, the stability between Ang-1 and Ang-2 may be predominantly helpful with respect to the endothelial activation and disease severity [10]. Furthermore, our findings are in agreement with another finding in which the ratio of Ang-2 : Ang-1 was found to be the lowest in healthy control subjects, and higher levels were seen in severe malaria [25]. A significant decrease (*p* < 0.05) was obtained for Ang-2 : Ang-1 ratio between severe malaria and uncomplicated malaria group and also with control group but was not seen between uncomplicated malaria and healthy control group. This finding additionally associates the dysregulation of angiopoietins as a contributing factor in malaria infections which might ultimately lead to endothelial dysfunction and disease severity. This further suggests that the sense of balance between Ang-1 and Ang-2 may help provide information about the state of the endothelium and the severity of the disease. Also, a study similar to our finding found that Ang-2 : Ang-1 levels were higher in patients with severe than nonsevere *Plasmodium falciparum* malaria and better indicated the severity of the disease [10]. Though a high level of parasitaemia is commonly associated with an increased risk of severe malaria, it may also occur in individuals with relatively low parasitaemia [24]. The ROC analysis also established the usefulness of Ang-2 and Ang-2: Ang-1 as good biomarkers of malaria and its severity. Although, Ang-1 did not show a good discriminating power, its analysis is necessary to determine the Ang-2 and Ang-1 ratio.

There was a significant increase in WBC counts in those with severe malaria infection compared with either uncomplicated malaria or control subjects, although no significant difference was observed between the WBC based on age groups in both severe and uncomplicated malaria. This increased level of white blood cells could be as a result of white cells that have been activated to fight the infection.

The results of this study showed a significant decrease in the red blood cell count groups, while an increased number of RBC was seen in healthy control group which is similar to a previous study [26]. The reduced levels of red blood cell in severe and uncomplicated malaria group might be as a result of the sequestration of red blood cell that occurs during *Plasmodium falciparum*-induced malaria infection and even during therapy. The findings are also similar to a previous work where reduced platelet counts was seen in the severe malaria group compared with the values obtained in the uncomplicated malaria group, while higher levels of platelet count was observed in the control group [27]. The reduced levels of Ang-1 in malaria subjects attest to the fact that Ang-1 is secreted by platelet granules, and thus its concentration in serum samples is dependent on the number of circulating platelets [28]. The low level of platelets supports the decreased Ang-1 level since Ang-1 is said to be stored in platelets, and thrombocytopenia is said to be an underlining characteristic of severe malaria infection.

Subsequently, each study group was categorized into three age groups which consisted children between the ages of 0 and 5 years, 6 and 10 years, and 11 and 15 years. These groupings showed that many (63.3%) of the children in severe malaria group were between the ages of 0 and 5 years compared with the other age group. This finding is similar to the reports of other researchers who stated that the incidence of severe malaria infection is higher in children between ages of 0 and 5 years of age compared with other ages due to the presence of little or no immunity to infection [29, 30]. Also, a significant increase in Ang-1 and Ang-2 levels was found in children between 11 and 15 years in severe malaria infection compared with other age groups. While these might be as a result of difference in hormonal and genetic makeup of individuals due to age variation, the reason for these findings is unknown as this grouping has not yet been well studied by previous researchers. However, it is still worth noting that the age group 11–15 years' subjects with severe malaria infection had higher levels of Ang-2 than Ang-1 which may be harmful to the subjects.

## 5. Conclusion

This study showed that subjects with severe malaria had a significant increase of Ang-2 and Ang-2 : Ang-1 ratio but presented with a significant decrease level of Ang-1, RBC, and platelets compared with uncomplicated malaria and control subjects who had higher levels of Ang-1, RBC, and platelets and lower levels of Ang-2 and Ang-2 : Ang-1 ratio. These results suggest that the dysregulation of angiopoietins may be involved in the pathogenesis of severe malaria. Consequently, the findings of this study suggest that Ang-1 and Ang-2 may be used as biomarkers to determine the severity of malaria infection.

## Figures and Tables

**Table 1:** Angiopoietin levels, red blood cells, and platelets in malaria and control subjects.

Parameters	Severe *n* = 30	Uncomplicated *n* = 40	Control *n* = 22	*F* value	*p* value
Ang-1 (pg/ml)	3238 ± 2667	4936.75 ± 2304	8808 ± 5166	18.439	0.000^*∗∗*^
Ang-2 (pg/ml)	7551 ± 3948	4530.87 ± 2534.70	4938 ± 3605	7.735	0.001^*∗∗*^
Ang-2 : Ang-1 ratio (pg/ml)	3.76 ± 3.27	1.13 ± 1.05	0.5590 ± 0.16	19.989	0.000^*∗∗*^
WBC (×10^9^/L)	12.70 ± 3.31	8.03 ± 1.51	8.16 ± 2.24	38.142	0.000^*∗∗*^
RBC (×10^12^/L)	3.46 ± 0.95	4.31 ± 0.69	5.03 ± 0.89	23.288	0.000^*∗∗*^
Platelet (×10^6^/L)	148.17 ± 25.50	193.80 ± 18.76	209.50 ± 26.82	52.726	0.000^*∗∗*^

^*∗*^Significant at *p* < 0.05. ^*∗∗*^Significant at *p* < 0.01.

**Table 2:** Levels of angiopoietins and hematological parameters according to age groups in uncomplicated malaria subjects.

Parameter	0–5 years *n* = 15	6–10 years *n* = 16	11–15 years *n* = 9	*F* value	*p* value
WBC (×10^9^/L)	7.81 ± 1.63	7.86 ± 1.49	7.48 ± 2.33	0.147	0.864
RBC (×10^12^/L)	4.03 ± 0.65	4.51 ± 0.72	4.42 ± 0.63	2.044	0.144
Platelet (×10^6^/L)	188.20 ± 15.68	201 ± 22.16	190.11 ± 13.57	2.186	0.127
MP density (parasites/*μ*l)	18.20 ± 10.28	15.98 ± 5.98	10.30 ± 4.98	2.973	0.063
Ang-1 (pg/ml)	5663 ± 3024	4362 ± 1927	4748 ± 1106	1.293	0.287
Ang-2 (pg/ml)	4499 ± 2355	4654 ± 3294	4362 ± 1094	0.038	0.963
Ang-2 : Ang-1 ratio	0.87 ± 0.38	1.46 ± 1.93	0.95 ± 0.30	0.957	0.393

**Table 3:** Levels of angiopoietins and hematological parameters according to age groups in subjects with severe malaria.

Parameter	0–5 years *n* = 19	6–10 years *n* = 8	11–15 years *n* = 3	*F* value	*p* value
WBC (×10^9^/L)	12.54 ± 3.52	12.71 ± 3.58	13.03 ± 1.55	0.030	0.971
RBC (×10^12^/L)	3.54 ± 0.86	3.05 ± 0.94	4.00 ± 1.50	1.320	0.284
Platelet (×10^6^/L)	143.68 ± 24.10	151.75 ± 20.44	167 ± 44.30	1.208	0.314
MP density (parasites/*μ*l)	100 ± 36.00	109 ± 34.00	74 ± 19	1.075	0.355
Ang-1 (pg/ml)	2057 ± 1817	4267 ± 2288	7973 ± 2003	13.255	0.000^*∗∗*^
Ang-2 (pg/ml)	6074 ± 2614	9102.70 ± 5096	12770 ± 1555	6.206	0.006^*∗∗*^
Ang-2 : Ang-1 ratio	4.70 ± 3.77	2.33 ± 1.02	1.64 ± 0.27	2.370	0.113

^*∗*^Significant at *p* < 0.05. ^*∗∗*^Significant at *p* < 0.01.

**Table 4:** Area under the ROC curve.

Parameters	Area	Std. Error	95% CI	*p* value
WBC (×10^9^/L)	0.9117	0.03592	0.8412 to 0.9821	<0.0001
RBC (×10^12^/L)	0.7813	0.05837	0.6668 to 0.8957	<0.0001
Platelet (×10^6^/L)	0.9258	0.03578	0.8557 to 0.9960	<0.0001
MP density (parasites/*μ*L)	0.9967	0.003595	0.9896 to 1.004	<0.0001
Ang-1 (pg/ml)	0.5046	0.07118	0.3650 to 0.6441	0.9480
Ang-2 (pg/ml)	0.8100	0.05434	0.7035 to 0.9165	<0.0001
Ang-2 : Ang-1 ratio	0.7233	0.06501	0.5959 to 0.8508	0.001479

## Data Availability

The data may be obtained from the corresponding author for researchers who meet the criteria for access to confidential data.
